# Supplementary material for: Purified F-ATP synthase forms a Ca2+-dependent high-conductance channel matching the mitochondrial permeability transition pore
Source: Nat Commun. 2019 Sep 25;10:4341. doi: 10.1038/s41467-019-12331-1 (PMC6761146; doi:10.1038/s41467-019-12331-1)
Supplement: Supplementary file 1 — Supplementary Information [file 41467_2019_12331_MOESM1_ESM.pdf]

Supplementary Information for

Purified F-ATP synthase forms a Ca^{2+}-dependent high-conductance channel matching the mitochondrial permeability transition pore

Andrea Urbani[1], Valentina Giorgio[1,2], Andrea Carrer[1], Cinzia Franchin[1,3], Giorgio Arrigoni[1,3], Chimari Jiko[4], Kazuhiro Abe[5], Shintaro Maeda[6], Kyoko Shinzawa-Itoh[7], Janna F.M. Bogers[8], Duncan G.G. McMillan[8], Christoph Gerle[9,10], Ildikò Szabò[2,11], Paolo Bernardi[1,2]

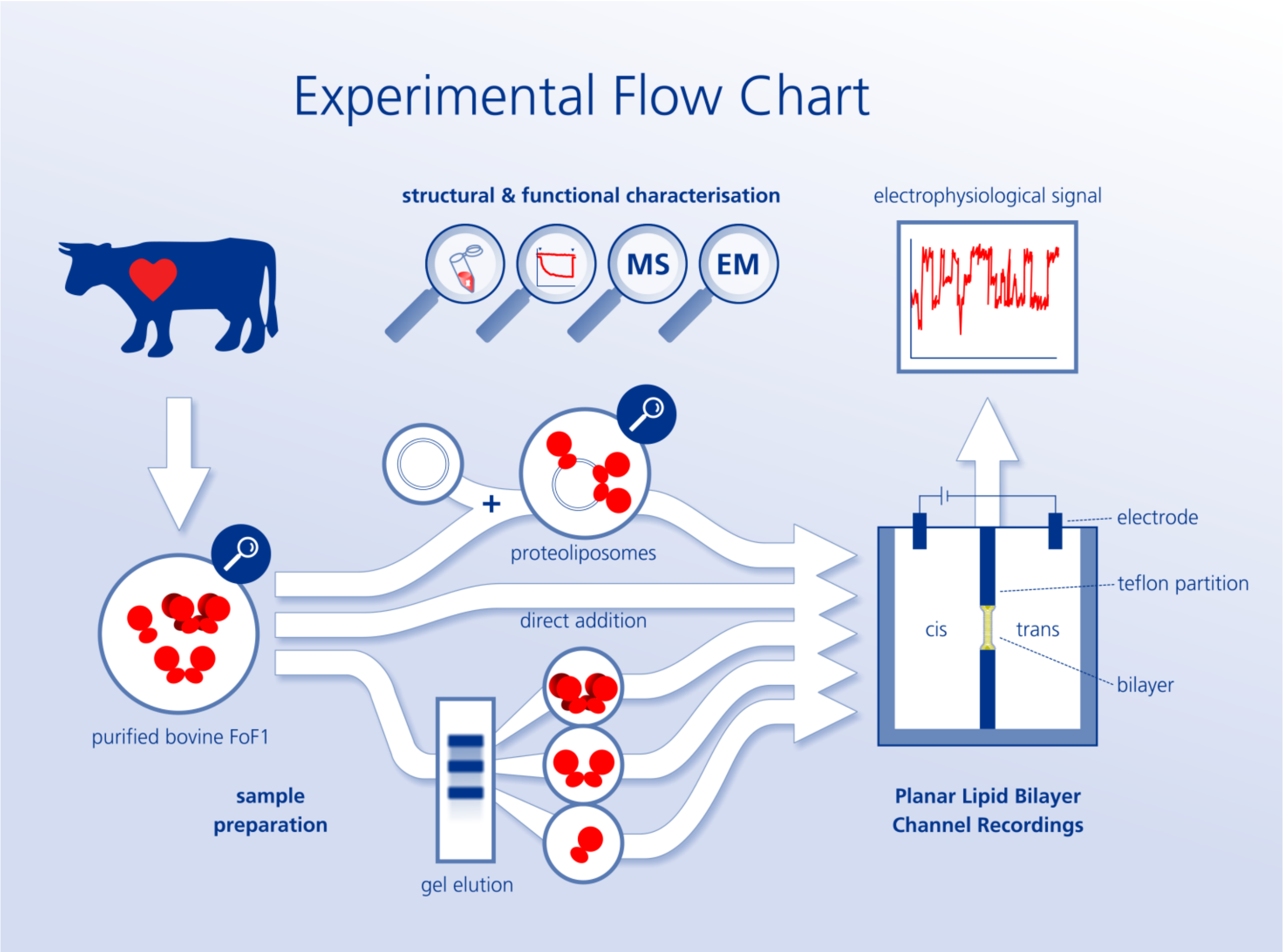

Supplementary Figure 1 Flow chart illustrating the logic of the experimental design. Preparations of bovine heart F-ATP synthase were analyzed for their structural and functional properties by biochemical assays, enymatic assays, mass spectrometry (MS) and electron microscopy (EM). The protein complex was (i) incorporated into liposomes, where its functional properties were tested, and then added to a planar lipid bilayer (top channel); (ii) directly added to the planar lipid bilayer (middle channel); or (iii) separated by BN-PAGE into monomers, dimers and oligomers which were then eluted and individually added to the planar lipid bilayer (bottom channel).

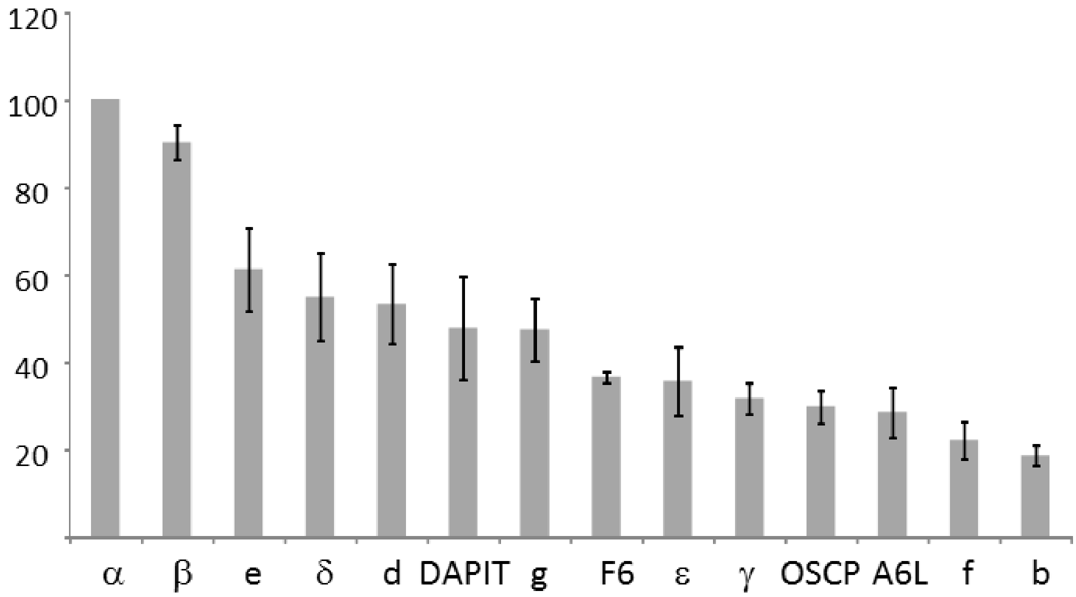

Supplementary Figure 2 Estimated abundance of F-ATP synthase subunits relative to α (%)

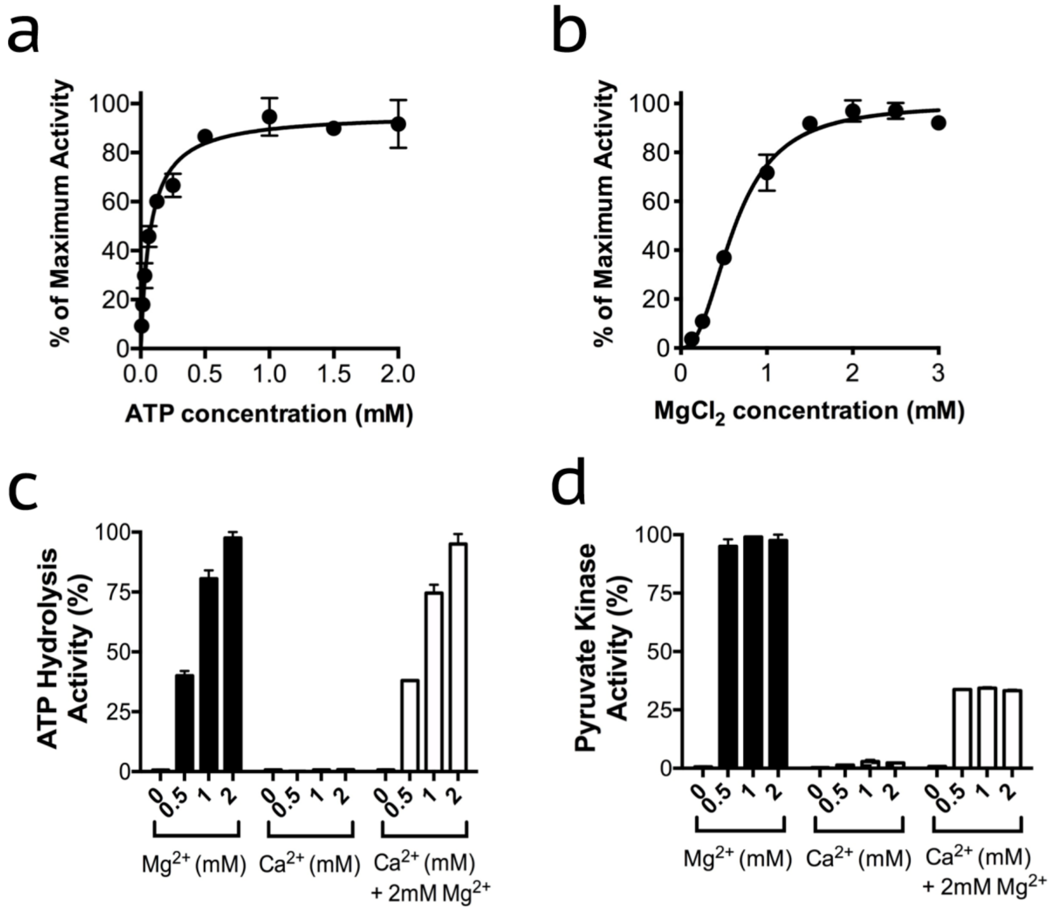

Supplementary Figure 3 Effect of ATP, Mg^{2+} and Ca^{2+} on ATPase and pyruvate kinase activities. ATP hydrolysis was measured at pH 7.4 using the ATP-regenerating assay described under "Methods" with increasing amounts of ATP (**a**), Mg^{2+} (**b**), and Mg^{2+} and Ca^{2+} as indicated (**c, d**). One hundred percent activity was 4.6 U/mg protein. Results shown were obtained in three independent experiments; the SEM is shown. The source data are provided as a Source Data file.

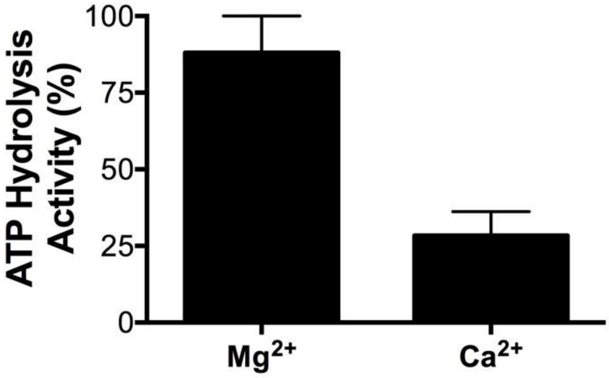

Supplementary Figure 4 Activation of ATP hydrolysis by Mg^{2+} and Ca^{2+}. ATP hydrolysis was measured at pH 7.4 using an inorganic phosphate assay described under "Methods". One hundred percent activity was 4.2 U/mg protein. Results shown were obtained from three independent experiments; the SEM is shown. The source data are provided as a Source Data file.

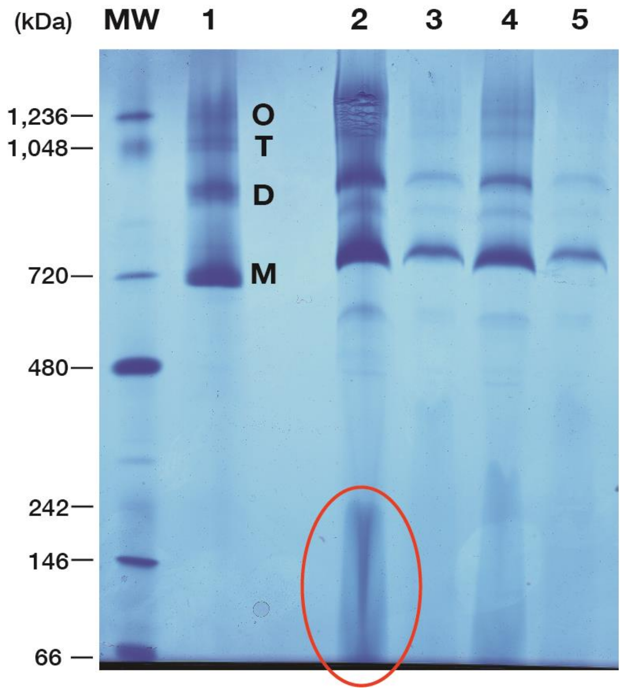

Supplementary Figure 5 BN-PAGE of digitonin re-solubilized proteoliposomes. To assess whether the liposome-reconstituted bovine F-ATP synthase retained its oligomeric state, proteoliposomes were re-solubilized by addition of digitonin and subjected to BN-PAGE electrophoresis. Lane MW: molecular weight markers (Da); Lane 1: bovine F-ATP synthase before reconstitution (30 µg); M, monomers, D, dimers, T, tetramers, O, oligomers. Lane 2: spun-down proteoliposomes; Lane 3: supernatant; Lane 4: spun-down proteoliposomes; Lane 5: supernatant. Encircled in red is the smear-like feature typical of lipids in native gels. Re-solubilization with digitonin and subsequent BN-PAGE did not cause a significant decrease of oligomeric complex. The total amount of bovine F-ATP synthase employed in the liposome reconstitution experiment for lanes 2,3 and lanes 4,5 were 30 µg and 15 µg, respectively.

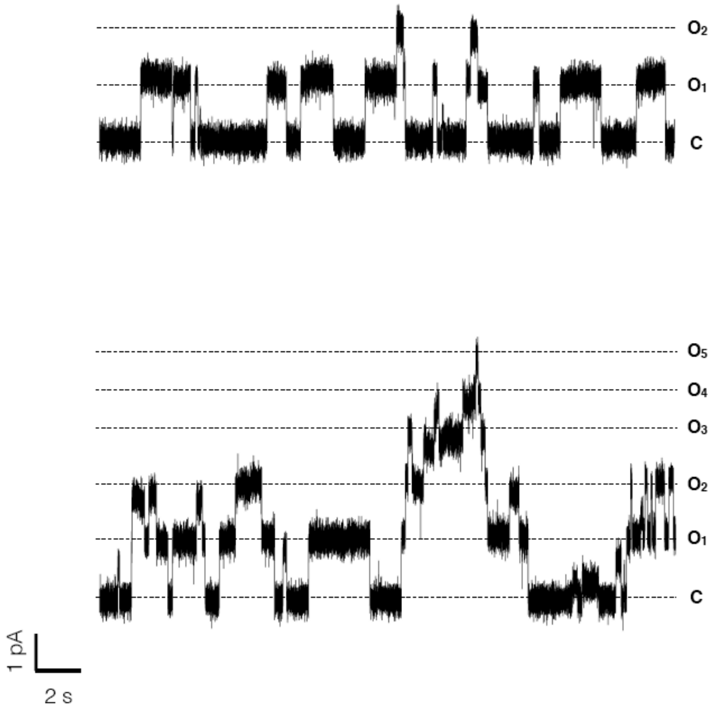

Supplementary Figure 6 Gramicidin D single channel currents in the planar lipid bilayer. Proteoliposomes were prepared with gramicidin D, added to the *cis* side and channel activity in the planar lipid bilayer was monitored. Representative recordings of current traces obtained after about 50 min are reported, showing the presence of two (upper trace) and five (lower trace) channels; V_{cis} = 60 mV.

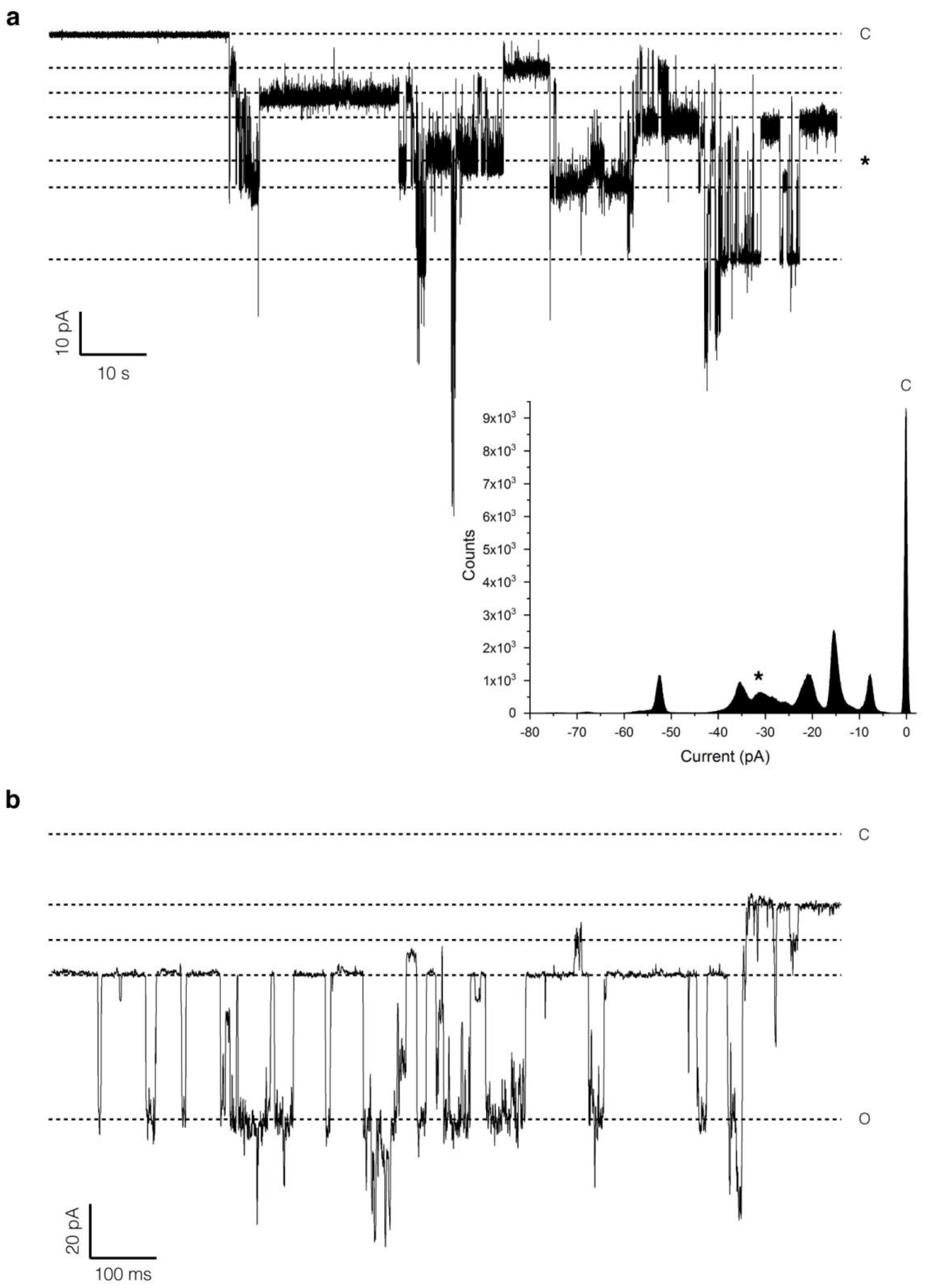

Supplementary Figure 7 High-conductance openings of bovine F-ATP synthase channels. Currents were elicited by 3 mM Ca^{2+}, 0.1 mM Bz-423 and 0.2 mM PhAsO after direct addition of the purified preparation of F-ATP synthase; V_{cis} = -80 mV. **a**, High conductance openings of bovine F-ATP synthase channels from the closed state. Discrete current levels can be seen in the amplitude histogram shown below the current tracings. Dotted lines indicate the 0 pA current level, corresponding to the closed state of the channel (C) and various subconductance states; the most populated open state is marked with a star. **b**, Representative high-conductance openings of bovine F-ATP synthase channels from another experiment, with conductance values ranging up to the typical full conductance of the mammalian PTP (~1.3 nS). Dotted lines indicate the 0 pA current level, corresponding to the closed state of the channel (C), and several open substates. The 1.3 nS conductance is indicated (O).

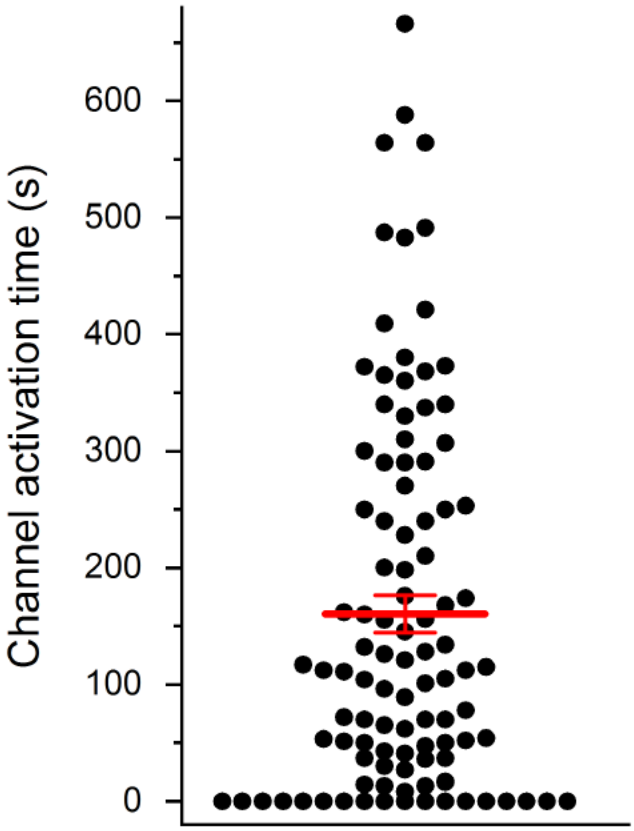

Supplementary Figure 8 Time to activation of F-ATP synthase. Purified F-ATP synthase preparations were added directly to the planar lipid bilayer. After up to 5 minutes (a period of time during which currents were never observed) 0.15 mM Bz-423 was added followed by sequential Ca^{2+} additions giving final concentrations of 0.10, 0.30, 0.66, 1.00 and 3.00 mM. No openings were observed unless Ca^{2+} was added. The plot reports the time at which the first channel opening occurred, and each point refers to an individual experiment (n = 103). The average time-to-opening was 2.68 min, and error bar refers to the SEM. The source data are provided as a Source Data file.

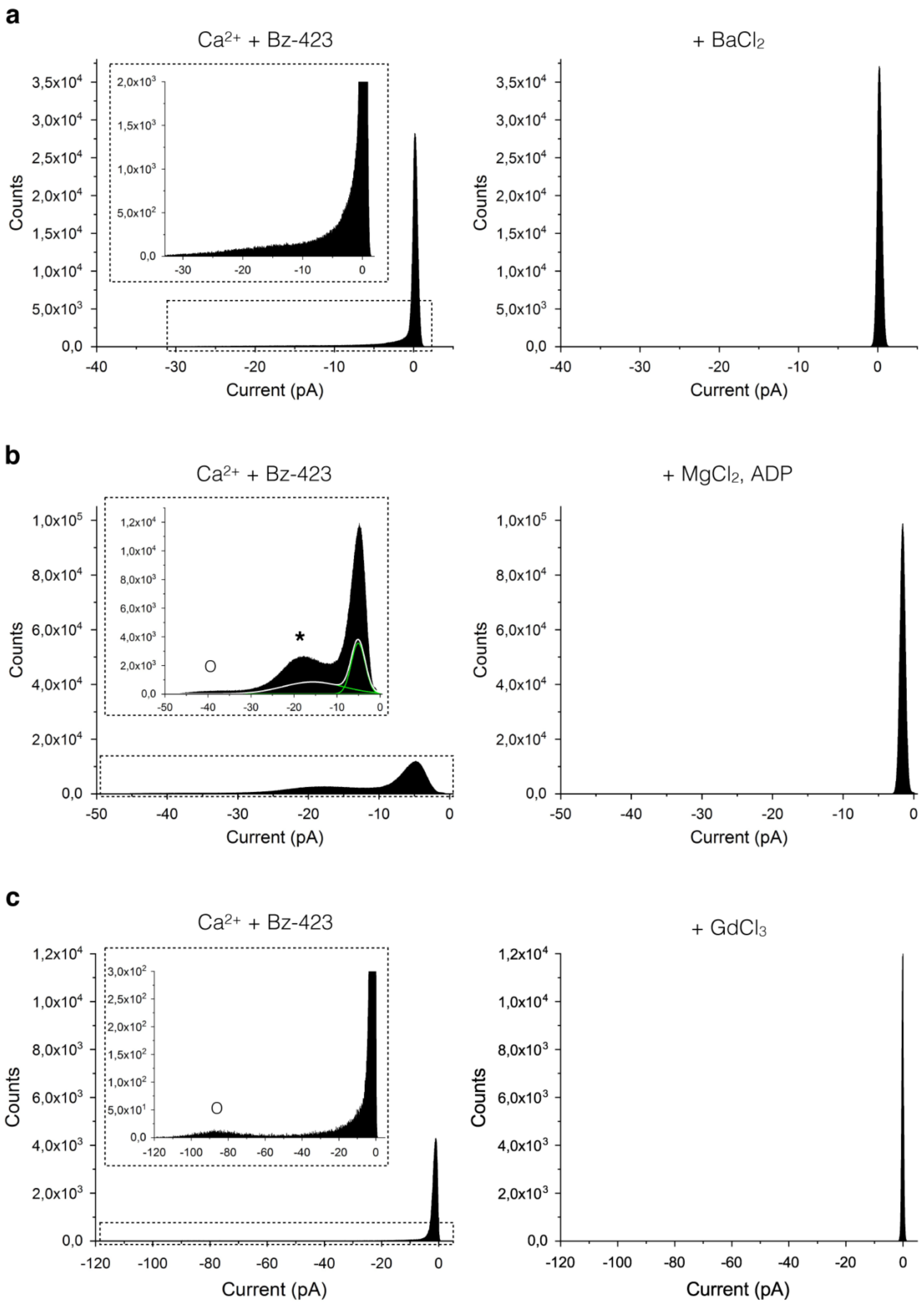

Supplementary Figure 9 Inhibition of F-ATP synthase channels by PTP/MMC inhibitors. **a,b,c**, Amplitude histograms showing current distribution of F-ATP synthase channels from 60 s-long recordings taken before and after the addition of PTP/MMC inhibitors, as shown in Supplementary Figure 3c, 3d and 3e, respectively.

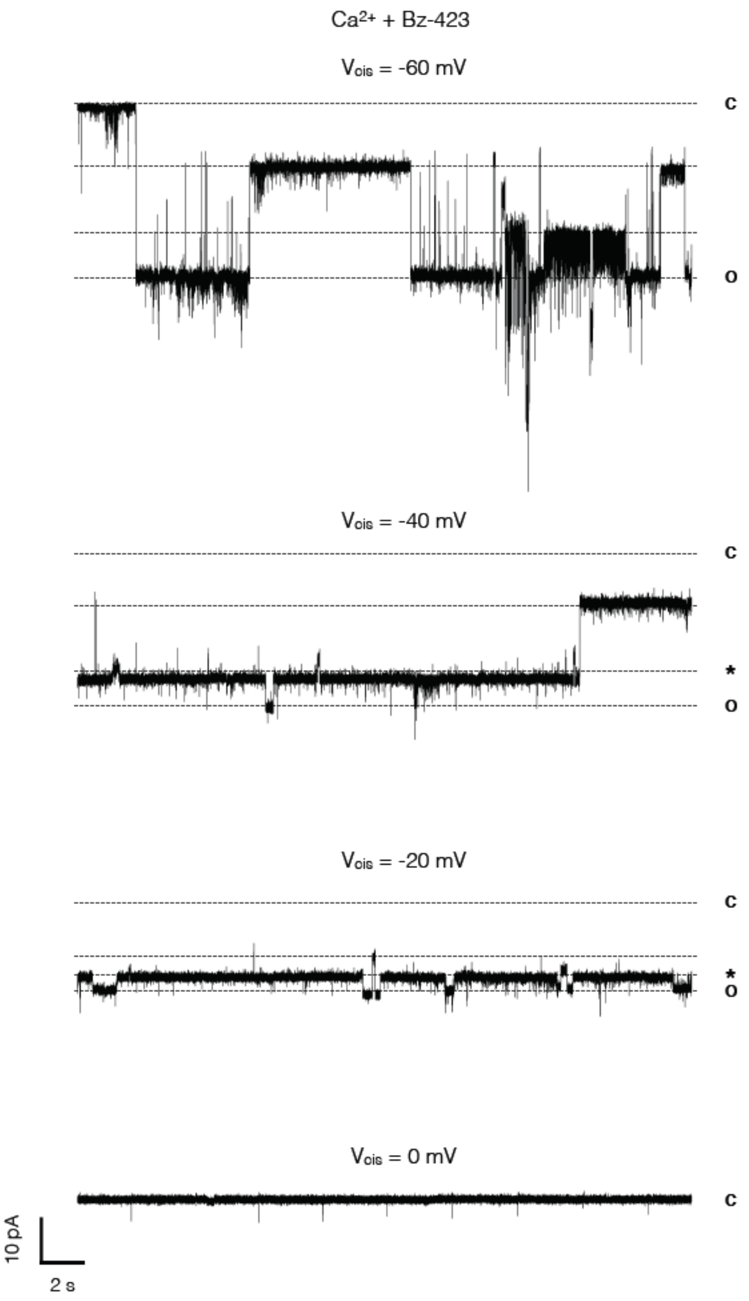

Supplementary Figure 10 Current recordings of F-ATP synthase channels at various applied voltages. Channel activity was elicited after direct protein addition followed by 3 mM Ca^{2+} and 0.1 mM Bz-423; V_{cis} was as indicated on each trace. Dotted lines indicate the 0 pA current level, corresponding to the closed state of the channel (C) and various subconductance states; the most populated open states are marked by stars.

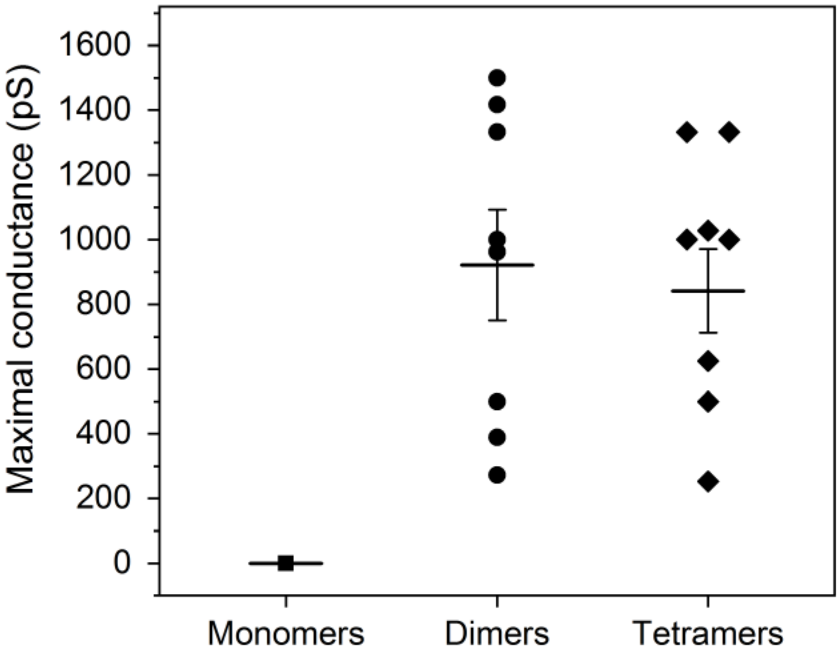

Supplementary Figure 11 Maximal conductance of channels formed by gel-eluted F-ATP synthase. Monomers, dimers, and tetramers of F-ATP synthase were eluted from BN-PAGE gels and reconstituted into planar lipid bilayers (1-2 μg of protein for each experiment); channel activity was assessed in the presence of Ca^{2+} up to 3 mM and 0.15 mM Bz-423 in the *cis* side, as shown in Supplementary Figure 4c. Maximal unitary conductance was calculated for each experiment and is reported on the graph. Mean and SEM are reported in the figure. No statistically significant differences were found between dimers (n = 8) and tetramers (n = 9) (Mann-Whitney test, p = 0.80) while difference between monomers (n = 7) and dimers, and monomers and tetramers, was significant (p ≤ 0.05). The source data are provided as a Source Data file.

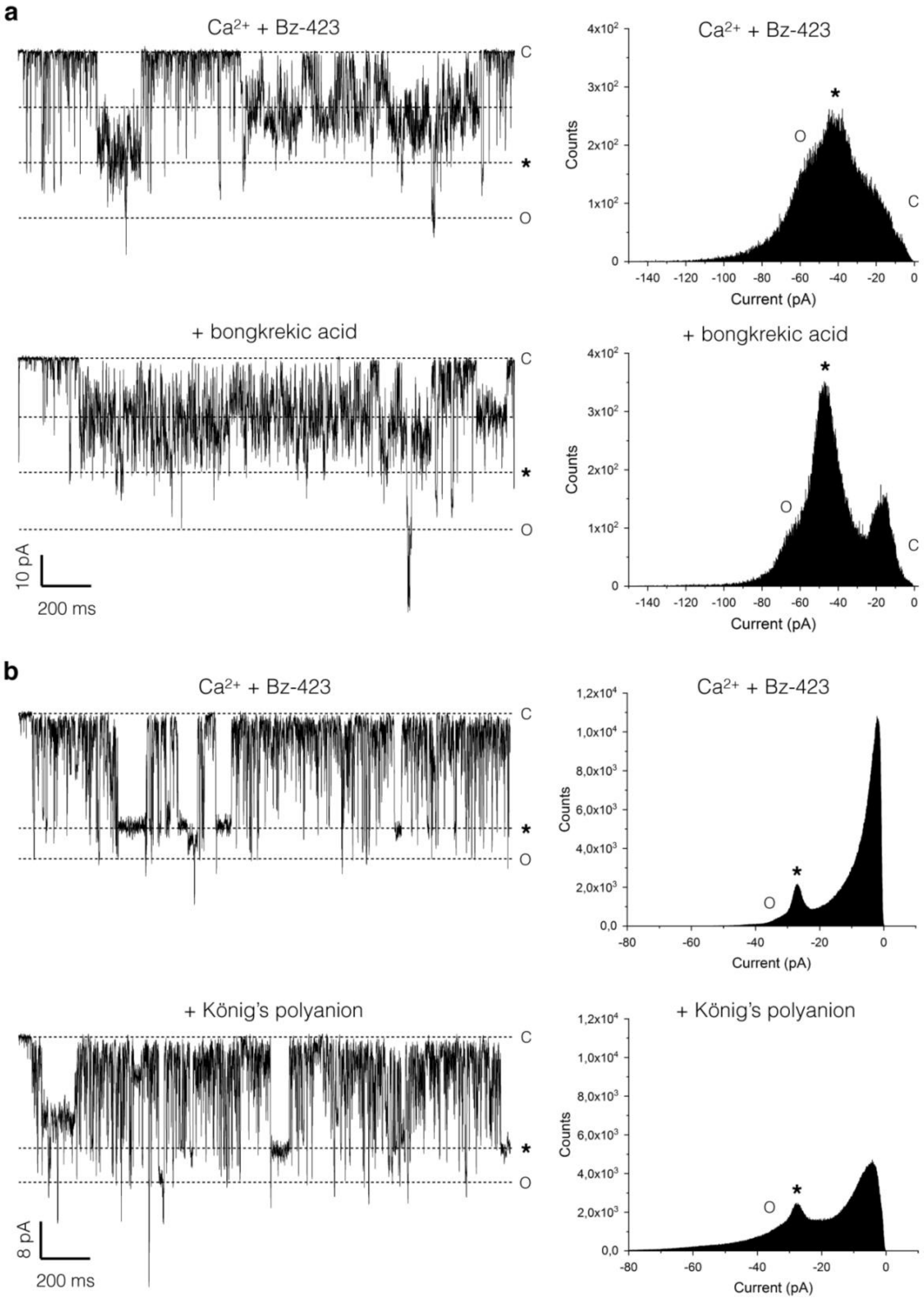

Supplementary Figure 12 Lack of effect of ANT and VDAC inhibitors on F-ATP synthase channel activity. **a**, Left: representative F-ATP synthase current traces elicited after addition of 3 mM Ca^{2+} and 0.15 mM Bz-423 before and after the addition of 10 µM bongkrekic acid, a selective ANT inhibitor, to both sides of the membrane; V_{cis} = -60 mV. Right: amplitude histograms corresponding to 60 s of recording. **b**, Left: representative F-ATP synthase current traces elicited after addition of 1 mM Ca^{2+} and 0.15 mM Bz-423 before and after the addition of 1 µg/ml of König's polyanion, a selective VDAC inhibitor, to both sides of the membrane; V_{cis} = -60 mV. Right: amplitude histograms corresponding to 60 s of recording.

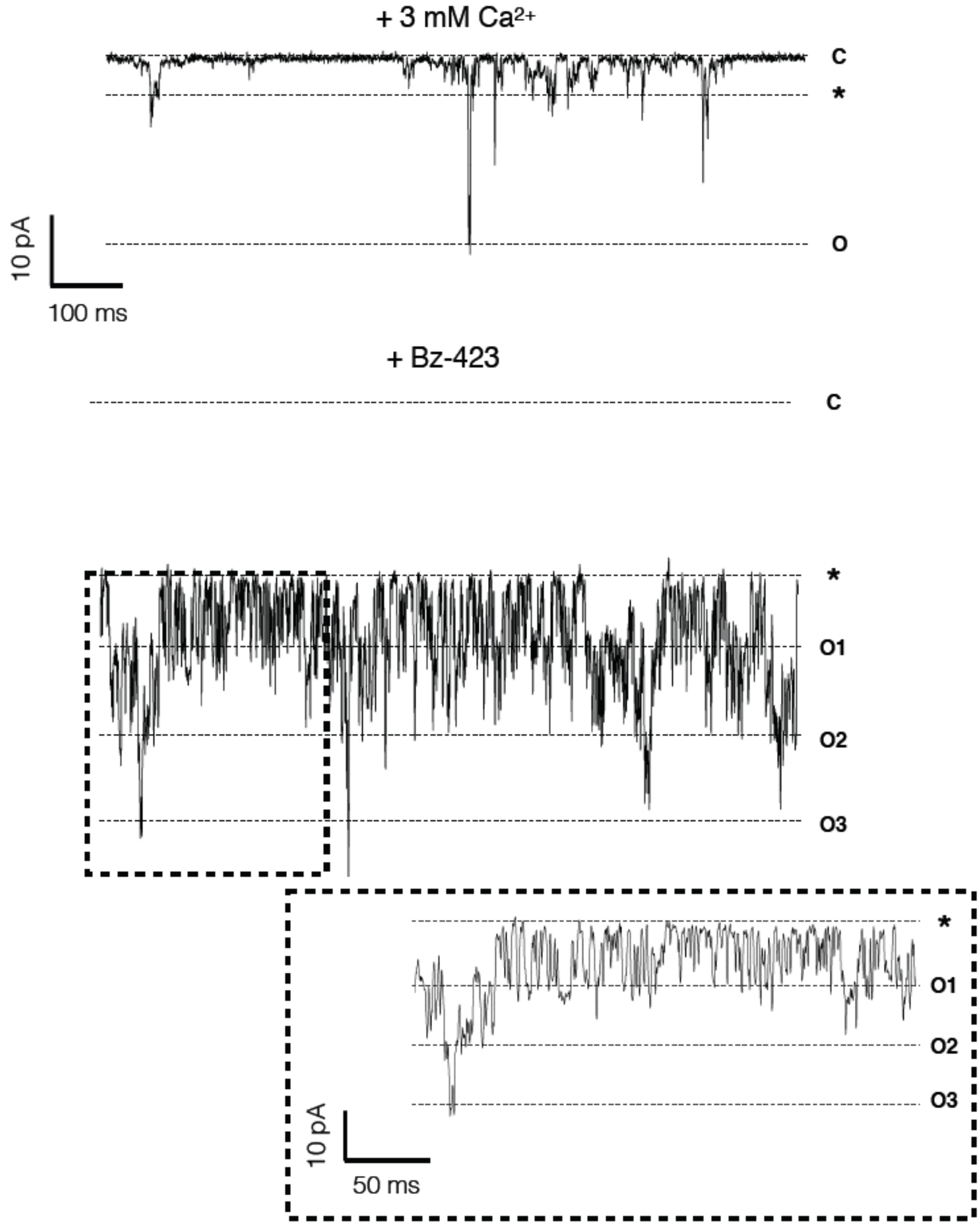

Supplementary Figure 13 Current traces elicited after addition of Ca^{2+} alone or after further addition of Bz-423. V_{cis} = -60 mV. Following the addition of 3 mM Ca^{2+} (top) the maximal open state (O) corresponded to a conductance of about 400 pS. Addition of 0.15 mM Bz-423 during the same recording increased the maximal open state (O3) to a conductance of about 1 nS. Additional subconductance states (O1,O2) were observed. Boxed area, the recording is also shown with an expanded time scale. The most frequent subconductance states are marked with an asterisk and the experiment reports one representative recording of five.

lower MW subunits as detected by MALDI-TOF/MS			
subunit	measured MW	theoretical MW	deviation (ppm)
δ	15060.42	15064.93	-299.2
g*	11325.52	11328.30	-244.9
f	10206.62	10209.00	-232.9
F6	8957.26	8958.09	-92.4
e	8188.63	8189.47	-102.3
A6L	7964.27	7964.00	34.2
c*	7650.63	7650.05	76.5
**6.8PL**	6833.72	6.834.08	-52.3
**DAPIT**	6302.95	6303.39	-69.4
ε	5651.36	5651.67	-54.4

*acylated/trimethylated

Supplementary Table 1 Lower molecular weight subunits as detected by MALDI-TOF MS. The same fractions used for clear native PAGE and SDS-PAGE were analyzed by MALDI-TOF MS in the mass range of 5,000 to 12,000 dalton. All expected subunits including the easily lost subunits 6.8PL and DAPIT were detected.

Protein	Gene name	Subunit	Average Abundance (% relative to α subunit)	S.E.M.
ATP synthase subunit alpha, mitochondrial	*ATP5A1*	α	100.00	
ATP synthase subunit beta, mitochondrial	*ATP5B*	β	90.22	4.04
ATP synthase subunit e, mitochondrial	*ATP5I*	e	61.15	9.50
ATP synthase subunit delta, mitochondrial	*ATP5D*	δ	54.86	10.03
ATP synthase subunit d, mitochondrial	*ATP5H*	d	53.20	9.21
Up-regulated during skeletal muscle growth protein 5	*USMG5*	DAPIT	47.70	11.62
ATP synthase subunit g, mitochondrial	*ATP5L*	g	47.36	7.18
ATP synthase-coupling factor 6, mitochondrial	*ATP5J*	F6	36.53	1.31
ATP synthase subunit epsilon, mitochondrial	*ATP5E*	ε	35.58	7.73
ATP synthase subunit gamma, mitochondrial	*ATP5C1*	g	31.60	3.47
ATP synthase subunit O, mitochondrial	*ATP5O*	OSCP	29.75	3.73
ATP synthase protein 8	*MT-ATP8*	A6L	28.48	5.74
ATP synthase subunit f, mitochondrial	*ATP5J2*	f	22.04	4.28
ATP synthase F(0) complex subunit B1, mitochondrial	*ATP5F1*	b	18.51	2.24
ADP/ATP translocase 1	*SLC25A4*	ADP/ATP Transl 1	1.26	0.17
Cytochrome c oxidase subunit NDUFA4	*NDUFA4*	CytC Oxidase Sub4	0.67	0.16
SLC25A12 protein	SLC25A12	Asp/Glut Carrier	0.47	0.05
Phosphate carrier protein, mitochondrial	*SLC25A3*	Phosphate Carrier	0.30	0.04
Uncharacterized protein	SLC25A13	Asp/Glut Carrier	0.13	0.01
Voltage-dependent anion-selective channel protein 2	*VDAC2*	VDAC2	0.07	0.01
Voltage-dependent anion-selective channel protein 1	*VDAC1*	VDAC1	0.06	0.01
Sodium/potassium-transporting ATPase subunit alpha	*ATP1A1*	Na^+/K^+ transporting ATPase	0.03	0.01
Calcium-transporting ATPase	*ATP2A2*	Ca^{2+} ATPase	0.02	0.01

Supplementary Table 2 Quantitative analysis of the protein composition of the F-ATP synthase preparation by LC-MS/MS. The estimated abundance was obtained using the iBAQ parameter from 6 independent measurements and is reported as percentage of the F-ATP synthase α subunit. Note that subunit a, c and 6.8PL, which were identified by MALDI-TOF MS (Extended Data Table 1), were not identified in this data set. Subunit a was found with a single peptide (see Extended Data Table 3) and therefore filtered out during the analysis. Under our analytical conditions, upon trypsin digestion subunit c produces a single potentially detectable peptide (DIDTAAK). This has been detected only twice in its original form as a mono-charged species (www.peptideatlas.org), and was excluded by our analytical settings since the instrument method was limited to the fragmentation of multiply-charged ions. The 6.8 kDa mitochondrial proteolipid (6.8PL) was repeatedly detected with up to 3 tryptic peptides in samples extracted from blue native gels but was not detected in this set of analyses.

```
function PSA(pot)
% pot : intermembrane potential (must be < 0)
warning('off');d=cd;[PathName]=uigetdir(d,'Select your data
folder');cd(PathName);d=dir(PathName);str = {d.name};
[s,v] = listdlg('PromptString','Select files:','SelectionMode','multiple','ListString',str);I = [];
if isempty(s),
   return
else
   figure,
   for i=1:length(s),
      name=d(s(i)).name;
      try
         [p,f,ext,ver] = fileparts(name);
      catch
         [p,f,ext] = fileparts(name);
      end
      try
         if isempty(ext),
            return;
         end
         if strcmp('.abf',ext)==1,
            try
               data(i) = vreadingabf(fullfile(PathName, name));
            catch
               load(name);
            end
         elseif strcmp('.mat',ext)==1,
            load(name);
         else
            return;
         end
      catch
         return;
      end
      y = double(data(i).Data(1:data(i).Npn,1)');v = double(data(i).Data(1:data(i).Npn,2)');
      x=(0:1:data(i).Npn-1)*data(i).DeltaT*1e-6;Fs = 1/(x(2)-x(1));Y = y(v>pot-5 & v<pot+5);
      Y(1:Fs*5) = [];Y(end-Fs*5:end) = [];V = v(v>pot-5 & v<pot+5);
      if isempty(V)==1,
         return
      elseif length(V)*Fs < 5,
         return
      end
      V(1:Fs*5) = [];V(end-Fs*5:end) = [];
      try
         idx1 = find(V>pot+4);
         Y = [Y(1:idx1(1)-Fs*1) Y(max(idx1)+Fs*5:end)];V = [V(1:idx1(1)-Fs*1) V(max(idx1)+Fs*5:end)];
      catch
      end
      try
         idx1 = find(V<pot-4);Y = [Y(1:idx1(1)-Fs*1) Y(max(idx1)+Fs*5:end)];
         V = [V(1:idx1(1)-Fs*1) V(max(idx1)+Fs*5:end)];
      catch
      end
      Y(Y<-200) = [];Y(Y>5) = [];Y = Y(1:end-Fs*1);X = (0:1:length(Y)-1)/Fs;
      [f,Y_Power] = PowerSpectrum(X,Y,0);Y_Power(f<51 & f>49) = 0;
      Y_Power(f2<5) = 0;Y_Power(f2>500) = [];Y_Power = Y_Power.*length(Y_Power);
      T1 = f(2)-f(1);A(i) = 0;
      for j=1:length(Y_Power)-1,
         A(i) = A(i)+(y2(j)+y2(j+1))*T1/2;
      end
   end
end
figure, plot(A,'.-','MarkerSize',50), ylabel('Power Spectrum Area'), xlim([0.5 length(A)+0.5])
saveas(gcf, 'PSA', 'fig')
```

Supplementary Table 3 Algorithm used to calculate the Power Spectrum Area

```
function Po_Imean_Imax(pot)
% pot : intermembrane potential
warning('off');d=cd;[PathName]=uigetdir(d,'Select your data
folder');cd(PathName);d=dir(PathName);str = {d.name};
[s,v] = listdlg('PromptString','Select files:','SelectionMode','multiple','ListString',str);I = [];
if isempty(s),
   return
else
   figure,
   for i=1:length(s),name=d(s(i)).name;
      try
         [p,f,ext,ver] = fileparts(name);
      catch
         [p,f,ext] = fileparts(name);
      end
      try
         if isempty(ext),
            return;
         end
         if strcmp('.abf',ext)==1,
            try
               data(i) = vreadingabf(fullfile(PathName, name));
            catch
               load(name);
            end
         elseif strcmp('.mat',ext)==1,
            load(name);
         else
            return;
         end
      catch
         return;
      end
      y = double(PATCH(i).Data(1:pippo(i).Npn,1)');v = double(PATCH(i).Data(1:pippo(i).Npn,2)');
      x=(0:1:pippo(i).Npn-1)*pippo(i).DeltaT*1e-6;Fs = 1/(x(2)-x(1));rum = std(y(1:Fs*1));
      Y = y(v>pot-5 & v<pot+5);Y(1:Fs*5) = [];Y(end-Fs*5:end) = [];V = v(v>pot-5 & v<pot+5);
      if isempty(V)==1,
         return
      elseif length(V)*Fs < 5,
         return
      end
      V(1:Fs*5) = [];V(end-Fs*5:end) = [];
      try
         idx1 = find(V>pot+4);Y = [Y(1:idx1(1)-Fs*1) Y(max(idx1)+Fs*5:end)];
         V = [V(1:idx1(1)-Fs*1) V(max(idx1)+Fs*5:end)];
      catch
      end
      try
         idx1 = find(V<pot-4);Y = [Y(1:idx1(1)-Fs*1) Y(max(idx1)+Fs*5:end)];
         V = [V(1:idx1(1)-Fs*1) V(max(idx1)+Fs*5:end)];
      catch
      end
      Y_offset = min(abs(smoothing(Y,50)));Y = Y-Y_offset*pot/abs(pot);X = (0:1:length(Y)-1)/Fs;
      figure, plot(X,Y), title('Total current'), xlabel('Time (s)')
      nbin = 50;[ord,asc] = hist(Y,nbin);[val,pos] = find(ord>Fs*0.01);
      try
         if pot/abs(pot) < 0,
            Imax(i) = abs(asc(pos(1)));
         elseif pot/abs(pot) > 0,
            Imax(i) = abs(asc(pos(end)));
         end
      catch
         Imax(i)= 0;
      end
      Ttot = length(X);
      if rum<3,
         X(Y>-3*rum & Y<3*rum)=[];Y(Y>-3*rum & Y<3*rum) = [];
      else
         X(Y>-2 & Y<2)=[];Y(Y>-2 & Y<2) = [];
      end
      Y = Y(1:end);
      X = X();
      if isempty(Y)==1,
         Y = 0;
      elseif length(Y) < Fs/10,
         Y = 0;
      end
      I(i).curr = Y;I(i).tempo = X;
      try
         YY = smoothing(Y,20);
      catch
         YY = Y;
      end
      Q(i) = abs(sum(YY))/length(Y);D(i) = std(Y);Topen = length(Y);Att(i) = Topen/Ttot*100;
      if Q(i) ==0,
         Imax(i) = 0;
      end
   end
end
figure, plot(0:length(s)-1,Q,'.-','MarkerSize',50), title('Mean Current'), ylabel('I_mean
(pA)'),xlim([-0.5 length(s)+0.5])
saveas(gcf, 'MeanCurrent', 'fig')
figure, plot(0:length(s)-1,Imax,'.-','MarkerSize',50), title('Maximal Current'), ylabel('I_max
(pA)'),xlim([-0.5 length(s)+0.5])
saveas(gcf, 'MaximalCurrent', 'fig')
figure, plot(0:length(s)-1,Att,'.-','MarkerSize',50), title('Open probability'), ylabel('Po %'),xlim([-0.5
length(s)+0.5])
saveas(gcf, 'OpenProbability', 'fig')
```

Supplementary Table 4 Algorithm used to calculate Po, G_{mean} and G_{max}

Supplementary Notes

The mass spectrometry proteomics data have been deposited to the ProteomeXchange Consortium via the PRIDE[1] partner repository with the dataset identifier PXD015108, Username: reviewer39520@ebi.ac.uk, Password: mHV18awM.

Supplementary reference

1. Perez-Riverol, Y. *et al.* The PRIDE database and related tools and resources in 2019: improving support for quantification data. *Nucleic Acids Res.* **47**, D442-D450 (2019).
